# Prevalence of Non-psychiatric Comorbidities in Pre-symptomatic and Symptomatic Huntington's Disease Gene Carriers in Poland

**DOI:** 10.3389/fmed.2020.00079

**Published:** 2020-03-11

**Authors:** Daniel Zielonka, Grzegorz Witkowski, Elzbieta A. Puch, Marta Lesniczak, Iwona Mazur-Michalek, Mark Isalan, Michal Mielcarek

**Affiliations:** ^1^Department of Public Health, Poznan University of Medical Sciences, Poznan, Poland; ^2^First Department of Neurology, Institute of Psychiatry and Neurology, Warsaw, Poland; ^3^Department of Human Evolutionary Biology, Adam Mickiewicz University, Poznan, Poland; ^4^Department of Histology and Embryology, Poznan University of Medical Sciences, Poznan, Poland; ^5^Department of Life Sciences, Imperial College London, London, United Kingdom; ^6^Imperial College Centre for Synthetic Biology, Imperial College London, London, United Kingdom

**Keywords:** huntington's disease, peripheral pathology, comorbidities, multimorbidity, pre-symptomatic stage, symptomatic stage

## Abstract

Huntington's disease (HD) is monogenic neurodegenerative disorder caused by CAG expansions within the Huntingtin gene (*Htt*); it has a prevalence of 1 in 10,000 worldwide and is invariably fatal. Typically, healthy individuals have fewer than 35 CAG repeats, while the CAG expansions range from 36 to ~200 in HD patients. The hallmark of HD is neurodegeneration, especially in the striatal nuclei, basal ganglia and cerebral cortex, leading to neurological symptoms that involve motor, cognitive, and psychiatric events. However, HD is a complex disorder that may also affect peripheral organs, so it is possible that HD patients could be affected by comorbidities. Hence, we investigated the prevalence of comorbid conditions in HD patients (pre-symptomatic and symptomatic groups) and compared the frequency of those conditions to a control group. Our groups represent 65% of HD gene carriers registered in Poland. We identified 8 clusters of comorbid conditions in both HD groups, namely: musculoskeletal, allergies, cardiovascular, neurological, gastrointestinal, thyroid, psychiatric, and ophthalmologic. We found that HD patients have a significantly higher percentage of co-existing conditions in comparison to the control group. Among the 8 clusters of diseases, musculoskeletal, psychiatric, and cardiovascular events were significantly more frequent in both pre- and symptomatic HD patients, while neurological and gastrointestinal clusters showed significantly higher occurrence in the HD symptomatic group. A greater recognition of comorbidity in HD might help to better understand health outcomes and improve clinical management.

## Introduction

Huntington's disease (HD) is the most common hereditary neurodegenerative disorder and is caused by CAG trinucleotide repeat expansions within the Huntingtin gene (*Htt*) (1), whose products are constitutively expressed in many tissues and organs (2–5). Whereas, healthy subjects have fewer than 35 CAG repeats, HD patients range from 35 up to ~200, and repeat size correlates with the age of disease onset (1). HD is invariably fatal and a recent study showed that prevalence is higher than previously thought, with 1 in 400 being at risk of developing HD (6). There is currently no treatment that targets the molecular cause of the disease, although several approaches are in development that specifically target the pathological mutant Huntingtin gene (HTT) (7); these include novel gene therapy tools such as synthetic zinc finger proteins (8). The best known feature of HD is neurodegeneration, and this is particularly widespread in the striatal nuclei, basal ganglia and cerebral cortex, resulting in neurological symptoms that involve motor, cognitive, and psychiatric disturbances (1). The neurodegeneration leads to a wide-range of clinical features including personality changes, motor impairment, subcortical dementia and weight loss. These symptoms progress over the course of the disease, typically resulting in death after 15–21 years (7).

There is growing evidence that Huntington's disease (HD) is a multi-system disorder (9). Although HD is largely recognized as a neurodegenerative disease, there is a mounting body of data, based on murine models of HD, that the peripheral component to its pathology may also contribute to disease progression. For example, HD-related cardiomyopathy has been recently discovered in two HD mouse models (10–12), where HD hearts displayed typical characteristics of heart failure, including increased cardiac apoptosis, elevated cardiac interstitial fibrosis (10), and altered energy homeostasis and purine metabolism (13, 14). Furthermore, some of these pathological features were already present at the pre-symptomatic stage (15). Another typical peripheral pathology associated with HD onset is skeletal muscle atrophy (16). In HD mouse models, skeletal muscle showed an altered energy metabolism and purine metabolism (17), caused by a distorted transcriptional network architecture of key enzymes involved in these processes (18). This led to a progressive deficiency in the contractile characteristics of the hind limb muscles, tibialis anterior (TA) and extensor digitorum longus (EDL), followed by a significant loss of motor units. In addition, these functional muscle impairments were accompanied by an aberrant deregulation of contractile protein transcripts and their up-stream transcriptional regulators (17). On the molecular level, mitochondrial dysfunctions, Peroxisome Proliferator-Activated Receptor (PPAR) alpha signaling and Heat Shock Factor 1 (HSF1) activation were identified as major players in HD-related muscle pathology, both in pre- and clinical settings [reviewed in Zielonka et al. (16)]. One might therefore conclude that the pathological features of HD skeletal muscles share a number of similarities on the molecular level with those caused by cancer cachexia (19). These are manifested by an energy imbalance, depleted ATP levels and an increased level of inflammatory markers. Notably, peripheral HD pathology is not only restricted to the malfunction of striated muscles, since a number of pre- and clinical studies have shown alterations in liver function (20), thyroid activity (21), reduced bone mineralization (22), and adrenal gland function (23).

Currently, clinical assessment of HD patients is mainly restricted to specific measures related to symptoms such as: depression, anxiety, personality change, irritability, dementia, chorea, imbalance, and falls. So far little is known about other co-existing chronic conditions that might be associated with HD progression in humans. It is well-accepted that comorbidities have important implications for health outcomes, as well as clinical management, in virtually any type of complex disease. Importantly, investigating comorbid chronic diseases at an early or pre-symptomatic stage in HD patients might give an overview of pathogenesis, and may provide important insights regarding mechanistic pathways through which novel therapeutics might be targeted. Hence, in this study, we aim to identify any co-existing chronic diseases in HD patients at the pre-symptomatic and symptomatic stages of disease progression.

## Materials and Methods

### Study Design and Participants

The present study is based on surveys collected at two EHDN (European Huntington's Disease Network) sites in Poland (Poznan and Warsaw). The data presented here were collected during an annual visit of HD patients and their family members, as a part of the longitudinal *Enroll-HD* study in 2018. At each center, clinicians with long-standing experience in HD took a careful history and examined patients clinically; motor, psychiatric, and cognitive signs were scored using the Unified Huntington's Disease Rating Scale (24). HD patients with the UHDRS motor scale score >5 were enrolled into the group of symptomatic HD patients (374 patients), while those with UHDRS <5 were enrolled into the group of pre-symptomatic HD patients (95 patients). Family members of HD patients that were negative for HD mutations were enrolled into the healthy control group (74 individuals).

### Classification of Comorbid Clusters

All recorded chronic diseases were grouped into the cluster of diseases based on ICD-10 classification. Specific recorded chronic diseases that were grouped into comorbid clusters can be found below.

**Musculoskeletal:** rheumatoid arthritis, gonarthrosis, scoliosis, cervical or/and lumbosacral spondylosis, intervertebral disc degeneration, discopathy, back pain, osteoporosis; Injuries: joints dislocation, strain or luxation, fractures of various bones, injury of ligaments, tendons, muscles, head injury.
**Allergies**: allergy to drugs, atopic dermatitis, allergic rhinitis due to pollen, asthma.
**Cardiovascular**: arterial hypertension, atherosclerosis, acute myocardial infraction, chronic ischemic heart disease, cardiac arrhythmias, tachycardia, valves insufficiency, cerebral infarction, veins insufficiency, varicose veins of lower extremities.
**Neurological**: meningitis, sleep disorders, insomnia, epilepsy, nerve root and plexus disorder, carpal tunnel syndrome.
**Gastrointestinal**: gastric ulcer, duodenal ulcer, gastro-esophageal reflux disease, gastritis, acute appendicitis, inflammatory bowel disease, irritable bowel syndrome, colon polyps, rectal polyps, cholecystitis, cholelithiasis, gallbladder calculus.
**Thyroid**: hypothyroidism, hyperthyroidism, thyroid nodules, thyroid goiter.
**Psychiatric**: depression episodes or recurrent depressive disorder, bipolar affective disorders, dementia, organic mood disorders, obsessive-compulsive disorder, insomnia, neurosis.
**Ophthalmologic**: cataract, glaucoma, myopia, retinopathy.

### Statistical Analysis

Continuous variables were calculated as means ± standard deviations (Microsoft, Excel). Univariate logistic regression analysis was used to assess association between the number of comorbidities and all groups of patients and to calculate crude odds ratio (OR) within a 95% confidence interval (CI). *p*-values were calculated by ANOVA with Tukey's *post hoc* test (SPSS v20, IBM). All tests were two-sided and *p*-values below 5% were regarded as statistically significant.

## Results

### Study Population

The studied population consisted of 543 patients, of whom 374 were classified as symptomatic HD patients and 95 were classified as pre-symptomatic HD patients, based on the Unified Huntington's Disease Rating Scale (UHDRS) (Table 1). In addition, there was a cohort of 74 non-HD carriers that were enrolled into a control group. On average the symptomatic HD patients were 47.4 years old (men and women); the pre-symptomatic HD patients were younger, on average 34.4 years old (men) and 31.7 years old (women); the non-HD patients were of similar age to the pre-symptomatic HD patients at 35.4 years old (men) and 33.3 years old (women). HD progression occurs with age and it is anticipated that symptomatic HD patients will be on average older than the pre-symptomatic patients. For the symptomatic patients only the male cohort showed a statistically significant difference in comparison to healthy individuals (*p* < 0.046), while the female cohort was not statistically different from healthy individuals (*p* = 0.121) (One-way ANOVA). Importantly, the CAG repeat size between pre-symptomatic and symptomatic HD patients was not significantly different and on average the pre-symptomatic group showed 42.8 CAGs (men) and 42.3 (women), while the symptomatic HD group had 45.7 CAGs (men) and 44.0 CAGs (women) (Table 1). All patients in the control group were screened and confirmed as non-HD carriers.

**Table 1:** Cohort characteristics.

**Cohort characteristics**	**Men**	**Women**
**Number**	***N*** **(%)**	***N*** **(%)**
**Symptomatic** AGE Average (SD) CAG Average (SD)	153 (40.9) 47.4 (4.2) 45.7 (8.3)	221 (59.1) 47.4 (13.4) 44.0 (5.8)
**Pre-symptomatic** AGE Average (SD) CAG Average (SD)	41.0 (43.2) 34.4 (9.9) 42.76 (3.3)	54.0 (56.2) 31.7 (7.8) 42.33 (2.8)
**Healthy controls** AGE Average (SD) CAG Average (SD)	26 (35.1) 35.4 (8.9) 18.2 (3.1)	48 (64.9) 33.3 (10.1) 18.1 (3.2)

*CAG expansion. SD, Standard deviation*.

### Comorbidity Prevalence

**Figure 1:**
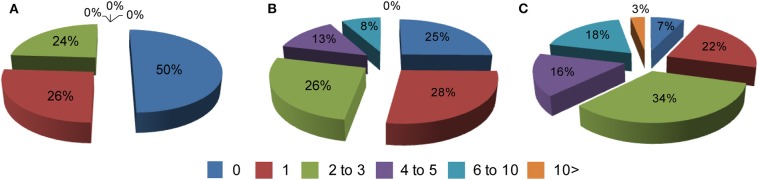
Percentage of comorbidities in: **(A)** a healthy control cohort (*n* = 74), **(B)** pre-symptomatic HD patients (*n* = 95), **(C)** symptomatic HD patients (*n* = 374) where 0–10 is a number of co-existing conditions in each group. Crude ORs of all registered comorbidities using univariate regression analysis for control group vs. symptomatic HD patients: 0 (Ors: 6.93; *p* < 0.001; 95% Cl: 3.98–12.07); 1 (Ors: 1.14; *p* = 0.63; 95% Cl: 0.66–1.99); 2–3 (Ors: 0.74; *p* = 0.27; 95% Cl: 0.42–1.28); 4–5 (Ors: 0.04; *p* < 0.05; 95% Cl: 0.002–0.691); 6–10 (Ors: 0.036; *p* < 0.05; 95% Cl: 0.0022–0.591); above 10 (Ors: 0.21; *p* = 0.29; 95% Cl: 0.0127–0.3794); control group vs. pre-symptomatic HD patients: 0 (Ors: 1.979; *p* < 0.025; 95% Cl: 1.089–3.95); 1 (Ors: 0.93; *p* = 0.85; 95% Cl: 0.48–1.82); 2–3 (Ors: 0.92; *p* = 0.82; 95% Cl: 0.47–1.82); 4–5 (Ors: 0.05; *p* < 0.05; 95% Cl: 0.003–0.88); 6–10 (Ors: 0.08; *p* = 0.08; 95% Cl: 0.0043–1.33); above 10 (Ors: 1.28; *p* = 0.9; 95% Cl: 0.025–65.37).

Comorbid conditions in all 3 groups were recorded based on an annual visit to HD clinics at two sites in Poland. Typically, patients were diagnosed with co-existing chronic conditions outside of the HD clinics, i.e., by primary or secondary care systems. Hence, the dataset analyzed here is based on fully diagnosed conditions. First, we assessed the frequency of comorbidities in all three study groups and we found that more than 10 co-existing chronic conditions can be found in symptomatic HD patients. Overall, there was a significant increase in the comorbid conditions between symptomatic HD patients and the control group for the data bins of 4–5 (*p* < 0.05) and 6 to 10 comorbidities (*p* < 0.05) (Figure 1). There was also a significantly higher percentage of symptomatic HD patients diagnosed with at least one chronic condition (*p* < 0.0001) in comparison to the control group. Similarly, there was a significantly higher percentage of pre-symptomatic HD patients with at least one comorbid condition (*p* < 0.05) in comparison to the control group. Moreover, a significant percentage of pre-symptomatic HD patients was diagnosed with 4 to 5 comorbid conditions (*p* < 0.05) in comparison to the non HD carriers (Figure 1).

We next examined the prevalence of the specific co-existing conditions in all 3 study groups. To simplify the presentation of comorbidities in our study, we grouped co-existing conditions into the following clusters of diseases, based on the ICD-10 classification: musculoskeletal, allergies, cardiovascular, neurological, gastrointestinal, thyroid, psychiatric, ophthalmologic, and without comorbidities (Table 2). We used univariate logistic regression analysis to assess the prevalence of each disease cluster in all studied groups (Table 3). We found that there were significantly less HD patients without any co-existing conditions: 25.3% (*p* < 0.05) (pre-symptomatic) and 7.5% (*p* < 0.0001) (symptomatic) in comparison to non-HD carriers (50%). There was a significantly higher percentage of symptomatic HD patients (31%) with musculoskeletal conditions (*p* < 0.001) in comparison to the control group (5.4%), while the pre-symptomatic HD cohort also showed a significantly higher percentage (20%) compared to the control (*p* < 0.05). The cardiovascular cluster of diseases showed a significantly higher prevalence in the HD patients: 25.1% (*p* < 0.002) (symptomatic) and 15.4% (*p* < 0.001) (pre-symptomatic), in comparison to the control group (1.3%). As expected, there was a significant increase in psychiatric events in the HD group: 61% (*p* < 0.0001) (symptomatic) and 20% (*p* < 0.05) (pre-symptomatic) in comparison to the control group (4%). In addition, there was an increase in the neurological conditions in the symptomatic HD group (12%; *p* < 0.05) but not in the pre-symptomatic HD group (10.5%; *p* = 0.16), in comparison to the non HD control patients (4%). The Gastrointestinal cluster showed a similar pattern, where 16.8% of symptomatic patients were diagnosed (*p* < 0.05), compared to only 7.4% of pre-symptomatic HD patients, and to 6.8% patients in the control group. Allergies, Thyroid and Ophthalmologic clusters of diseases did not show a statistically higher occurrence in the studied groups, even though there was a clear trend toward increase in the frequency of the Allergies and Ophthalmologic clusters, in both pre-symptomatic and symptomatic HD groups, in comparison to the control group. We have not observed a gender-specific increase in described comorbidities.

**Table 2:** Prevalence of clusters of diseases in the symptomatic HD, pre-symptomatic HD and control groups.

**Comorbidity**	**Non HD controls (%)**	**Pre-symptomatic HD (%)**	**Symptomatic HD (%)**
Without concomitant diseases	37 (50%)	24 (25.3%)	28 (7.5%)
Musculoskeletal	4 (5.4%)	19 (20%)	116 (31%)
Allergies	6 (8.1%)	16 (16.8%)	59 (15.8%)
Cardiovascular	1 (1.3%)	12 (12.6%)	94 (25.1%)
Neurological	3 (4%)	10 (10.5%)	45 (12%)
Gastrointestinal	5 (6.8%)	7 (7.4%)	63 (16.8%)
Thyroid	7 (9.5%)	8 (8.4%)	39 (10.4%)
Psychiatric	3 (4%)	19 (20%)	228 (61%)
Ophthalmologic	2 (2.7%)	7 (7.4%)	22 (5.9%)

**Table 3:** Crude Odds Ratios (ORs) for all comorbidities using univariate logistic regression analysis.

**Comorbidity**	**Control vs. Pre-symptomatic**			**Control vs. Symptomatic**		
	**Crude ORs**	***p*-value**	**95% CI**	**Crude ORs**	***p*-value**	**95% CI**
Without concomitant diseases	1.979	< 0.05	1.089–3.595	5.8393	<0.0001	3.63–10.14
Musculoskeletal	0.27	< 0.05	0.0882–0.8285	0.1524	<0.0003	0.0545–0.465
Allergies	0.481	=0.14	0.1796–1.2903	0.4494	=0.073	0.187–1.081
Cardiovascular	15.41	<0.001	1.9585–121.17	0.047	<0.002	0.0064–0.3427
Neurological	0.3851	=0.1583	0.1023–1.4497	0.2946	<0.05	0.0891–0.9738
Gastrointestinal	0.917	=0.886	0.2797–3.0058	0.3507	<0.05	0.1363–0.9023
Thyroid	1.1223	=0.8296	0.3896–3.2389	0.7931	=0.591	0.3413–1.8431
Psychiatric	0.2027	<0.05	0.0578–0.7111	0.0581	<0.0001	0.0181–0.1867
Ophthalmologic	0.3668	=0.2194	0.074–1.1879	0.4017	=0.229	0.0924–1.7461

*ORs and CI (Confidence interval) correspond to data in Table 2*.

## Discussion

Huntington's disease is a devastating neurodegenerative disorder characterized typically by progressive chorea and subcortical dementia (1). Motor impairments are typical characteristics of HD symptoms and in the late stage are characterized by dystonia, bradykinesia, and rigidity (1). HD is also described by a spectrum of various psychiatric features, as well behavioral abnormalities, including: depression, anxiety, irritability, apathy, obsessive-compulsive disorder (OCD) and psychosis (25). In addition, HD is manifested by a cognitive impairment that is associated with a significant memory decline, even at very early stage (26).

Although HD is primarily recognized as a neurological disorder, over the past decade there has been growing support for the idea that HD is a multi-system disorder that can lead to multi-organ failure (9). This conclusion is supported by studies of the biology of the HTT protein, as it has been shown to be uniformly expressed in various cell types in mammals (3). In fact, a genetic knock-out of *Htt* in mice led to a very early embryonic lethality at E8.5, which suggests that HTT may play an important role during embryonic development (27). The protein is also involved in multiple critical cellular processes such as transcription, protein trafficking and vesicle transport (28). Hence, one might conclude that peripheral pathology could be an important component of disease progression. However, there is still lack of clinical data supporting the findings from pre-clinical settings and in particular no previous study has attempted to identify and assess co-existing diseases at any stage of HD progression.

In order to investigate any possible comorbid conditions in HD, we analyzed over 500 surveys that were part of a longitudinal observational study by the EHDN grouping, where HD patients were asked about their diagnosed co-existing conditions. HD subjects were divided into the two groups of pre-symptomatic stage and symptomatic stage, so as to represent disease progression, while non-HD carriers (mainly family members of HD patients) were enrolled into the control group. First, we investigated the distributions of co-existing diseases in all 3 groups. We found that even pre-symptomatic HD patients had a significantly higher number of comorbid conditions in comparison to the healthy individuals. As expected, the symptomatic group of HD patients was characterized by a significantly lower percentage of subjects without any comorbidity (7%) in comparison to the control group (50%). Generally, HD patients have more comorbidities than controls and these increase as the disease progresses.

Next, we characterized the type of comorbidities in each group by aligning each disease unit to a specific cluster of diseases. As expected, the psychiatric cluster had the highest prevalence among symptomatic HD patients (61%) while pre-symptomatic HD patients showed only 20%. We identified two other clusters of comorbid conditions, namely musculoskeletal and cardiovascular, that showed sufficiently higher percentages of incidents to be statistically-significant in both HD groups, in comparison healthy controls. In the case of the musculoskeletal cluster, we observed that 60% of individuals in the pre-symptomatic group were affected by injuries and 40% by articulations of degenerative disorders. The symptomatic patients were affected as follows: 5% by rheumatoid diseases, 40% by articulations of degenerative diseases and 55% by injuries as a result of involuntary movements. The larger number of musculoskeletal conditions in symptomatic individuals may be partly explained by presence of involuntary movements that results in injuries and in abrasion of joints. However, chorea does not affect pre-symptomatic individuals therefore an intrinsic cellular function of mutant HTT should be considered as a source of peripheral pathology (29, 30). The gastrointestinal cluster increase in HD-manifesting individuals could be potentially related to problems with disturbed food biting, the consumption of medications and from the metabolic disturbances described in literature (31, 32).

The cardiovascular cluster showed the following frequencies for specific diseases in the pre-symptomatic HD cohort: 58% with arterial hypertension, 18% with cardiac arrhythmias, 8% with tachycardia, 8% with insufficiency of valves, and 8% with insufficiency of veins. The symptomatic HD cohort had the following percentage of specific diseases: 65% with arterial hypertension, 3% with atherosclerosis, 3% with acute myocardial infarction, 7% with chronic ischemic heart disease, 3% with cardiac arrhythmias, 2% with tachycardia, 3% with insufficiency of valves, 3% with cerebral infarction, 3% with insufficiency of veins, and 8% with varicose veins of lower extremities. Hence, it may be concluded that arterial hypertension was the most frequent chronic disease across both pre- and symptomatic HD patients. Supporting our findings, it has already been shown elsewhere that a cohort of early symptomatic patients (25.3%) showed significant contractile heart dysfunction (33). Abnormalities were manifested in several ways, including bradycardia and prolonged QTc interval and/or intra-ventricular conduction (33).

We also found that symptomatic HD patients had a significantly higher incidence of neurological and gastrointestinal clusters in comparison to the control group. A recently published study that compared the prevalence of non-motor symptoms between HD and PD patients revealed that HD patients had significantly a higher prevalence of dysphagia, constipation, bowel incontinence and fecal tenesmus than the PD group (34). Interestingly it has been reported that the R6/1 mouse model of HD had a significant difference in microbiota composition at 12 weeks of age. There was an increase in Bacteroidetes and decrease in Firmicutes in the microbiome. Moreover, there was an increase in microbial diversity in male R6/1 mice only, compared to controls. Importantly, dysbiosis coincided with impairment in body weight gain at 12 weeks of age (35). However, we did not see a large increase in thyroid-related conditions even though this might be expected after a previously published study showed a declining level of TSH (Thyroid Stimulating Hormone) with disease progression (21). Interestingly, other clusters of comorbidities, including Allergies and Ophthalmologic conditions, showed a trend toward an increase in both pre-symptomatic and symptomatic HD groups, approximately doubling in frequency in HD patients. Among ophthalmologic conditions, retinopathy occurred at frequencies of 36 and 15% in symptomatic and pre-symptomatic HD patients, respectively. We also observed that glaucoma was present in 30% of symptomatic cases and 23% of pre-symptomatic ones. Interestingly, 55% of cases were diagnosed with myopia in the HD symptomatic cohort, while only 27% of pre-symptomatic HD subjects developed this condition. In addition, there was a 14% frequency of cataracts in symptomatic HD patients whereas none of the pre-symptomatic HD patients developed this condition. Interestingly, HD mouse models showed early and progressive retinal degeneration with apparent photoreceptor cell loss (36). It is possible that the ophthalmologic conditions are underestimated in our studied groups of HD patients as our study is based on surveys rather than ophthalmologic examination.

Comorbid conditions may be considered to be an integral part of any complex disease, including neurodegenerative disorders, and by identification those co-existing conditions in the clinic, we may learn more about their progression. There is growing evidence that other complex neurodegenerative diseases are characterized by co-existing diseases. For example, Alzheimer disease has a number of comorbid conditions including vascular abnormalities, osteoporosis, thyroid diseases, and glaucoma (37). Parkinson's disease is also characterized by comorbidities such as: bone fractures, cancer, dementia, diabetes and stroke (38), with hypertension and diabetes being the most frequent (39).

Our current study has some limitations, since the presented results rely on surveys, and it is highly likely that HD patients might present with even more comorbidities if detailed clinical diagnostic testing were conducted. Considering the fact that a large number of patients were treated with antipsychotic drugs (quetiapine, olanzapine), it is certainly possible that this could contribute to an increase in metabolic disorders in HD patients. However, diabetes—often a side-effect of these medications—was not present in our cohort. Moreover, medication effects could be also excluded in the presymptomatic group where, to our knowledge, individuals were not treated with medications. Consequently, considering that a gastrointestinal cluster increase was also present in the pre-manifest group, when compared to controls (and subsequently in the manifest group), this demonstrates that medications were unlikely to have been the cause of this increase. One limitation of our study is the lack of data capture on medications, as potential confounders. On the other hand, such analyses could introduce false assumptions about our investigated cohort, with a lack of certainty that particular medication contributes to a particular observed disorder (it may or may not be attributable to a side-effect). Therefore, we avoid such assumptions and merely record comorbidities as objectively as possible. Nonetheless, we believe that our study will already have important implications in the health outcomes and clinical management of HD patients. It is known that environmental modifiers appear to have an impact both at the beginning and during the course of the disease. Indeed, there are currently many international initiatives aiming at assessing lifestyle factors (activity, nutrition, sleep, etc.), such as the Domino-HD project. We may therefore postulate that the higher prevalence of cardiological and musculoskeletal diseases can be both an effect of extra-CNS HD pathology, and also an influence on the progression of further disabilities, shortened lifespans, and decreases in the overall quality of life. Our experience with patients, referred to our sites from different centers, is that the numerous problems induced by HD itself may sometimes have a negative impact on the diagnostics and management of other comorbidities. The provision of strong evidence for increased prevalence of cardiological, musculoskeletal, and gastrointestinal comorbidities should lead to more intensive screening diagnostics and aggressive treatment of these entities and, in our opinion, is as important as the search for environmental modifiers of HD. Moreover, we expect that a better understanding of the molecular mechanisms underlying the occurrence of these comorbidities in HD will open the gates for a multidimensional approach to HD, incorporating comorbidities, and will provide new avenues for the advancement of individualized treatment in HD patients. Specifically, disease management should be concentrated on: (1) screening for the most common HD-concomitant disorders in pre-symptomatic and symptomatic HD individuals, (2) risk factor assessment tool development, and (3) prevention and treatment in early stages for those with a higher risk of a particular comorbid development. The discovery of new biomarkers and therapeutic targets seems to be the most important first step in this process of holistic management of HD with its comorbidities.

## Data Availability Statement

The datasets generated for this study are available on request to Daniel Zielonka (daniel.zielonka@gmail.com).

## Ethics Statement

All subjects gave their informed consent for inclusion before they participated in the study. The study was conducted in accordance with the Declaration of Helsinki, and the protocol was approved by the Ethics Committee at the Wielkopolska District Chamber of Physicians, Poznan, Poland, Decision No. 75/2016.

## Author Contributions

DZ, GW, EP, MI, and MM: conceived and designed the experiments. DZ, GW, EP, ML, and IM-M: performed the analysis. DZ, EP, ML, IM-M, and MM: analyzed the data. DZ, MI, and MM: wrote the manuscript.

### Conflict of Interest

The authors declare that the research was conducted in the absence of any commercial or financial relationships that could be construed as a potential conflict of interest.
